# High-throughput transcriptomics analysis of equipotent and human relevant mixtures of BPA alternatives reveal additive effects in vitro

**DOI:** 10.1007/s00204-025-04110-3

**Published:** 2025-06-18

**Authors:** Geronimo Matteo, Eunnara Cho, Marc Rigden, David C. Eickmeyer, Lauren M. Bradford, Matthew J. Meier, Andrew Williams, J. Christopher Corton, Carole L. Yauk, Ella Atlas

**Affiliations:** 1 Environmental Health Science and Research Bureau, Health Canada, Ottawa, Canada; 2 Department of Biology, University of Ottawa, Ottawa, Canada; 3 Center for Computational Toxicology and Exposure, US Environmental Protection Agency, Research Triangle Park, USA; 4 Department of Biochemistry, University of Ottawa, Ottawa, Canada

**Keywords:** Mixtures, Transcriptomics, Potency, Regulatory toxicology, Endocrine disruptor

## Abstract

While many jurisdictions have phased out use of bisphenol A (BPA), there is increasing exposure to mixtures of BPA alternatives. Like BPA, some alternatives perturb nuclear hormone receptors and are endocrine disruptors. We used high-throughput transcriptomics (HTTr) to evaluate the potency and modes of action of seven mixtures of BPA alternatives and their 12 individual components in breast cancer cells. Our aim was to explore whether alternatives present in mixtures act additively. MCF-7 cells were exposed to chemicals (0.001–50 µM) for 48 h and gene expression analysis was used to measure global and estrogen receptor alpha (ERα)-specific transcriptomic changes. Transcriptomic points of departure (tPODs) were derived using benchmark concentration (BMC) modelling. We first identified concentrations at which global transcriptional activity was robustly altered. Then, we applied a ERα transcriptomic biomarker to identify ERα agonists and predict ERα activation tPODs. We employed mixtures modeling to predict potency of BPA alternatives and test for additive effects in vitro. Ingenuity pathway analysis (IPA; Qiagen) was used to identify upstream regulators and canonical pathways from genes fitting BMCs. BPAF was the most potent individual chemical tested overall, followed by BPA and BPC. All seven mixtures had additive effects across all tPODs modeled. The ERα transcriptomic biomarker classified all mixtures as ERα activators along with several BPA alternatives. All mixtures and most individual components perturbed similar upstream regulators and pathways, suggesting common modes of action. These data support the value of HTTr in identifying additive effects and toxicological potency of mixtures in vitro.

**Supplementary Information:**

The online version contains supplementary material available at 10.1007/s00204-025-04110-3.

## Introduction

Bisphenol A (BPA) is an endocrine disruptor that affects nuclear hormone receptors including the estrogen and androgen receptors. BPA is primarily used in the manufacture of polycarbonate plastics, epoxy resins, and thermal paper; today, most people have measurable levels of BPA and its metabolites (Colorado-Yohar et al. 2021). Given empirical evidence demonstrating various adverse health effects, BPA has been increasingly replaced with alternative chemicals (Manzoor et al. 2022). Epidemiological data support that humans are exposed to multiple BPA alternatives (Gys et al. 2020; Kim et al. 2021; Moreno-Gómez-Toledano et al. 2023). Exposure primarily occurs orally as BPA and alternatives present as polymers degrade into monomers that can be ingested (Manzoor et al. 2022). Another common route of exposure is by contact with thermal paper (e.g., receipt paper), as BPA and alternatives like Pergafast 201 (P201) are used as developing agents (Björnsdotter et al. 2017). Despite their widespread use, many BPA alternative chemicals are data-poor, and there is mounting evidence that some may pose human health hazards.

Many BPA alternatives share common modes of action through ERα. For example, bisphenol AF (BPAF), bisphenol C (BPC), 4,4′-bisphenol F (4,4′-BPF), bisphenol B (BPB), bisphenol Z (BPZ), bisphenol AP (BPAP), and 4,4′-bisphenol S (BPS) are ERα agonists in vitro (Pelch et al. 2019; Keminer et al. 2020). Some, like BPAF, activate ERα at lower concentrations than BPA (Matteo et al. 2023). Importantly, evidence suggests that BPA alternatives have additive effects when present as mixtures in vitro. For example, a binary mixture containing BPA and BPAF behaved additively in ERα luciferase assay in T47D-KBluc cells (Bermudez et al. 2010). In HeLa cells expressing ERα, a ternary mixture of BPA, 4,4′-BPF, and BPS activated ERα at a concentration (0.43 μM) lower than predicted according to a logistic model (Park et al. 2020). Moreover, in a follow-up study using the same in vitro system, the authors found that binary and ternary mixtures of BPA, BPAF, BPB, BPC, BPE, and BPZ activated ERα in an additive manner (Lee et al. 2023). More research investigating the behavior of BPA alternatives and their interaction with ERα in mixtures is needed to better understand these effects.

High-throughput transcriptomics (HTTr) is increasingly being used for chemical potency assessment, mode of action analysis, and biologic read-across (Meier et al. 2024). We previously used HTTr to show that several BPA alternatives have comparable potencies to BPA in MCF-7 breast cancer cells and activate ERα at similar concentrations (Matteo et al. 2023). Beal and colleagues used a similar approach to screen 11 BPA alternatives also in MCF-7 cells (Beal et al. 2024). In MCF-10 breast epithelial cells, exposure to equimolar mixtures of BPA alternatives activated KEGG pathways associated with metabolism, ER protein processing, spliceosome function, and ubiquitin-mediate proteolysis (Mesnage et al. 2022). However, these mixtures were not assessed for additivity, synergy, or antagonism. These studies demonstrate the utility of HTTr in informing the toxicity of BPA alternatives for regulatory decision making (Reardon et al. 2023). Applying these methods to clarify how these chemicals interact in mixtures to alter endogenous gene expression would be highly valuable to advance knowledge.

Here, we used HTTr to determine if simple and complex mixtures of BPA alternatives behave additively in vitro. MCF-7 breast cancer cells were exposed to seven different mixtures of BPA alternative chemicals across a range of eight concentrations (0.001–50 µM), along with their 12 individual components, solvent control (0.1% DMSO), and a positive control (17β-estradiol, 1 and 10 nM) for 48 h in serum-free conditions. Templated Oligonucleotide Sequencing (TempO-Seq; BioSpyder Inc.) was used to quantify changes to the transcriptome and ERα-related gene sets. Transcriptomic biomarkers were used to assess if chemicals were ERα agonists and identify cellular stress conditions. Cytotoxicity of mixtures was assessed using a cell viability assay. BMC modeling was applied to gene expression data to derive transcriptomic points of departure (tPODs) that assess global, pathway, and ERα-specific transcriptomic changes. To test for additive effects, we applied a mathematical model previously used by our lab (Addicks et al. 2023) to predict the potency of BPA alternatives based on their individual components. Finally, genes fitting BMCs underwent pathway over-representation analysis to examine the toxicological mechanisms associated with the individual chemicals and their mixtures.

## Materials and methods

### Chemicals and mixtures

**Fig. 1:**
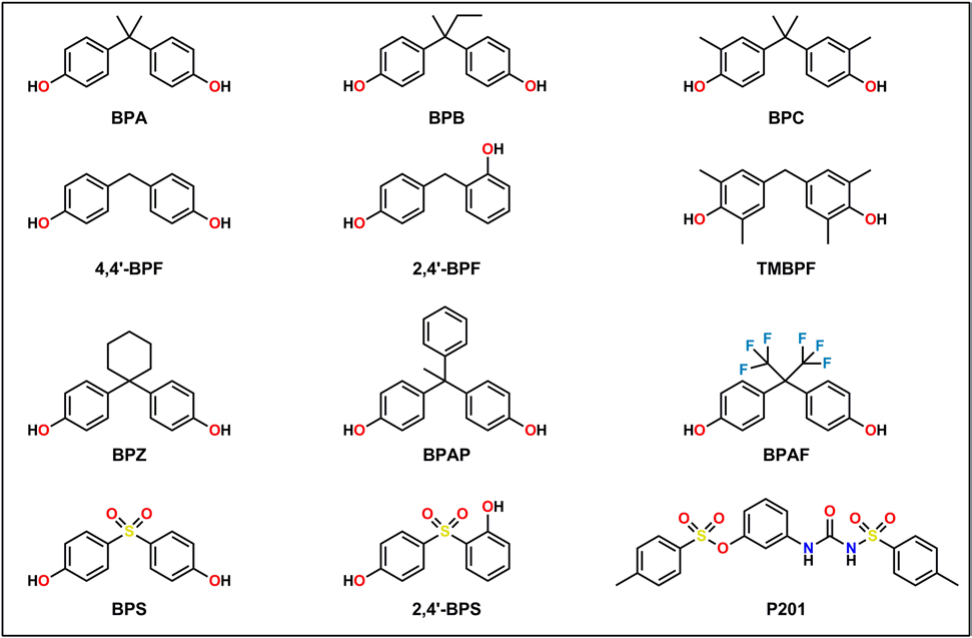
Chemical structure of bisphenol A (BPA) and alternative chemicals

Table 1 lists the chemicals used and their respective suppliers. All chemicals and mixtures (Fig. 1, Table 1) were dissolved in dimethyl sulfoxide (DMSO); the final DMSO concentration in exposure media was 0.1%. BPA and alternative chemicals were combined to form six different equimolar mixtures (Mix 1–Mix 6) and one complex (non-equimolar) mixture (Mix 7). Each mixture concentration (i.e., total chemical concentration) is the sum of the individual chemicals (e.g., 50-µM Mix 1 contains 25-µM BPA and 25-µM BPAF). The complex mixture (Mix 7) was made using the proportions of alternative chemicals detected in human urine (Gys et al. 2020). The final concentrations used in Mix 7 are the sum of the individual concentrations of each alternative chemical. Table 2 describes each mixture’s components and relative proportions of chemicals.

**Table 1:** List of chemicals and suppliers

Chemical	Acronym	CAS number	Supplier
Bisphenol A	BPA	80-05-7	Sigma-Aldrich Inc (St. Louis, MO, USA)
Bisphenol AF	BPAF	1478-61-1	
4,4′-Bisphenol F	4,4′-BPF	620-92-8	
Bisphenol S	BPS	80-09-1	
Bisphenol AP	BPAP	1571-75-1	
Estradiol	E2	50-28-2	
Dimethyl sulfoxide	DMSO	67–68-5	
Bisphenol B	BPB	77-40-7	
Bisphenol Z	BPZ	843-55-0	
Tetramethyl bisphenol F	TMBPF	5384-21-4	
Pergafast 201	P201	232938-43-1	Toronto Research Chemicals (Toronto, ON, Canada)
2,4′-Bisphenol S	2,4′-BPS	5397-34-2	
Bisphenol C	BPC	79–97-0	Accustandard (New Haven, CT, USA)
2.4′-Bisphenol F	2,4′-BPF	2467-03-0	TCI America (Portland, OR, USA)

**Table 2:** List of mixtures and relative proportions

Mixtures	Components	Proportions
M1	BPA:BPAF	Equimolar
M2	BPA:BPAF:BPC	Equimolar
M3	BPA:BPAF:BPC:4,4′-BPF	Equimolar
M4	BPA:BPAF:BPC:4,4′-BPF:BPAP	Equimolar
M5	BPA:BPAF:BPC:4,4′-BPF:BPAP:BPS	Equimolar
M6	BPA:BPAF:BPC:4,4′-BPF:BPAP:BPS:2,4′-BPF:P201:2,4′-BPS	Equimolar
M7	BPA:BPAF:4,4′-BPF:BPAP:BPS:BPB:BPZ	318:1:1789:6:320:3:13

### Cell culture

MCF-7 cells (ATCC, Manassas, Virginia) were cultured in 10-cm dishes (Corning Falcon No. 353003, Corning, New York) with Dulbecco’s modified Eagle’s medium (DMEM; Gibco, Thermo Fisher Scientific, Waltham, Massachusetts) containing 10% fetal bovine serum (FBS; Wisent Bioproducts, Saint-Jean Baptiste, QC, Canada), and 1% penicillin and streptomycin (P/S; Wisent), at 37 °C in a humidified atmosphere (5% CO_2_). For the experiment, cells were washed with phosphate-buffered saline (PBS) and phenol red-free DMEM supplemented with 5% charcoal–dextran stripped FBS (CD-FBS) was used to eliminate estrogens present in commercial medium (Atlas et al. 2003) for 48 h. Cells were then seeded into clear 96-well plates (Corning Falcon No. 353075) at a density of 2.5 × 10^4^ cells per well in phenol red-free DMEM supplemented with 5% CD-FBS. After 48 h, the medium was removed and replaced with phenol red-free DMEM containing 2% bovine serum albumin (BSA; supplier), 1% P/S, and chemicals of interest. BSA was used in place of CD-FBS in the exposure medium to further reduce the concentrations of estrogens and other hormones. Cells were exposed for 48 h, in quadruplicate, to BPA, the 12 alternative chemicals, the six equimolar mixtures of alternative chemicals at eight different concentrations (0.001, 0.01, 0.1, 0.5, 1, 5, 10, 50 µM), and one complex mixture. Concentrations for the complex mixture were 0.66, 1.3, 2.6, 5.2, 10.5, 21 µM. The experiment included cells exposed to 17β-estradiol (E2; 0.1, 1 nM) as a positive control and at least four plate-matched solvent controls (0.1% DMSO). The exposure timeframe was selected based on our previous study (Matteo et al. 2023). After exposure, cells were washed with PBS and lysed in situ using 2 × TempO-Seq lysis buffer diluted with an equal amount of PBS for TempO-Seq library building.

### Cell viability assay

To determine if chemical exposure decreased cell viability, MCF-7 cells were seeded into black 96-well plates (Corning Falcon No. 3603) and treated as described above. We tested all mixtures and a subset of chemicals (BPB and BPZ) that were not included in our previous study (Matteo et al. 2023). Cell viability was measured using the CellTiter-Blue Cell Viability Assay (Promega Corp, Madison, Wisconsin), as per the manufacturer’s instructions. This is a fluorometric assay that measures the metabolic capacity of cells based on resazurin reduction. Briefly, after 48-h exposure, CellTiter-Blue reagent was added to each well, and the plates were incubated (37 °C, 5% CO_2_) for 2 h. The fluorescence was read using an excitation wavelength of 560 nm and emission of 590 nm using a SpectraMax M2 (Molecular Devices LLC, San Jose, California). Fluorescence readings were expressed as a percent ratio to that of the DMSO control group. Cytotoxicity was defined as readings < 50% of the control.

### TempO-Seq library building and next-generation sequencing

Gene expression was measured using the TempO-Seq Human Whole Transcriptome v2.1 kit (BioSpyder Technologies Inc, Carlsbad, California) as per the manufacturer’s instructions and as previously described (Matteo et al. 2023). Cell lysates and positive technical controls (Human Universal Reference RNA—uhrRNA Agilent Cat No. 740000, Santa Clara, California, and Human Brain Total RNA brRNA—ThermoFisher AM7962, Waltham, Massachusetts), as well as no-cell negative controls (1 × TempO-Seq lysis buffer) were hybridized to the Detector Oligo (DO) Pool using an annealing kit for the whole human genome supplied by BioSpyder. The hybridization mixture was incubated for 10 min at 70 °C followed by a temperature gradient with a ramp rate of 0.5 °C/min to 45 °C over 50 min with a 16-h hold at 45 °C and then cooled to 25 °C. Nuclease digestion was employed to remove excess, unbound, or incorrectly bound DOs at 37 °C for 90 min. Amplification templates were generated by ligating DO pairs bound to adjacent target sequences for 1 h at 37 °C, followed by enzyme denaturation for 15 min at 80 °C. Amplification templates (10 µL) were pipetted into a 96-well PCR Pre-Mix and Primer plate supplied by BioSpyder and amplified using a CFX96 Real-Time PCR Detection System (Bio-Rad) to incorporate a unique sample index/tag sequence and the sequencing adaptors for each sample. The following PCR settings were used: 37 °C for 10 min, 95 °C for 1 min; 25 cycles of 95 °C for 10 s, 65 °C for 30 s, 68 °C for 30 s (with optical read for visual sample quality control [QC]); 68 °C for 2 min; and hold at 25 °C prior to library pooling and purification. For a list of attenuators used, see Supplementary File 1.

NucleoSpin Gel and PCR Clean-up kits were used to pool and purify labeled amplicons. Next-generation sequencing libraries were sequenced using a NextSeq 2000 High-Throughput Sequencing System (Illumina, San Diego, California), using 50 cycles from a 75-cycle high-throughput flow cell to achieve a median read depth of 2 million reads per sample. Data were processed as described below and reads were aligned to the BioSpyder TempO-Seq Human Whole Transcriptome probe set (22,537 probes over 19,687 genes).

### Data processing and quality control

Briefly, reads were demultiplexed from the BCL files and processed into FASTQ files using DRAGEN analysis v1.3.0 on the Illumina Basespace Sequence Hub (Illumina, Inc., San Diego, CA, USA). Processing and quality control of the FASTQ files, differential expression analysis, and exploratory statistical analyses were performed using the R-ODAF_Health_Canada pipeline (https://github.com/R-ODAF/R-ODAF_Health_Canada; accessed December 2024), which manages workflows using snakemake v8.0 (Mölder et al. 2021) and relies on scripts in R (v.4.3.2). The preprocessing steps use fastp v0.23.2 for trimming (Chen et al. 2018), STAR v2.7.8a (Dobin et al. 2013) to perform alignment of raw reads to the reference sequence and the qCount function of the QuasR R package (v1.30.0; Gaidatzis et al. 2015) to extract the feature counts from the aligned reads (BAM files) using features specified in a GTF file. A samples-by-probes count matrix was produced. Study-wide quality control was performed on the count matrix using several methods to measure consistency and remove low-quality samples, following the methods in Harrill et al. (2021) as a guideline. A cut-off of 0.1 for 1-Spearman’s *q* was used to remove samples that were not correlated with others in this study. Like Harrill et al. (2021), we also used a 10% cut-off of uniquely mapped reads as the number of target sequences (e.g., 100,000 reads to pass filter when the target is 1,000,000 for TempO-Seq experiments). We removed any samples outside of Tukey’s Outer Fence (3 × interquartile range) for: (1) the number of probes capturing the top 80% of the signal, and (2) the number of detected probes (those with at least five mapped reads). Samples with a Gini coefficient (a measure of inequality in distributions) > 0.95 were excluded. Samples removed by these criteria are listed in Supplementary File 2.

To create a matrix for biomarker analysis, individual pairwise contrasts for each concentration and each chemical tested were created to the respective 0.1% DMSO control samples for each plate. Following the recommendations set out by the Omics Data Analysis Frameworks for Regulatory application (R-ODAF) guidelines (Verheijen et al. 2022), genes were filtered for each contrast tested to include only those where 75% of at least one experimental group were above 0.5 counts per million (CPM), and spurious spikes were removed in which [max–median] of counts were less than [sum of counts]/[number of replicates + 1]. We used DESeq2 1.30.0 (Love et al. 2014) to estimate fold changes and normalize for library size within the TempO-Seq data. The ashr method was used to perform log_2_ fold change shrinkage (Stephens 2017). Data are available through the NCBI Gene Expression Omnibus with GEO accession GSE276869.

### Gene expression biomarker analysis

To determine if the BPA alternatives activate ERα or other pathways, the expression profile of each chemical-concentration tested relative to solvent control (derived from the analysis of gene sets with unadjusted *p* value < 0.05 and absolute linear fold change ≥ 1.2) was compared to several characterized biomarkers as previously described (Kupershmidt et al. 2010). The biomarkers included those that predict modulation of ERα (Corton et al. 2024a, b), nuclear factor erythroid 2-related factor 2 (NRF2; Rooney et al. 2020), heat shock factor 1 (HSF1: Cervantes and Corton 2021), metal-induced transcription factor 1 (MTF1; Jackson et al. 2020), aryl hydrocarbon receptor (AhR; Oshida et al. 2015), DNA damage (TGx-DDI; Li et al. 2019), histone deacetylase inhibition (TGx-HDACi; Cho et al. 2021), nuclear factor kappa B (NF-κB; Korunes et al. 2022), hypoxia inducible factor 1 (HIF1; unpublished data), and cellular proliferation (CP; Corton et al. 2024a, b). As well, we included an unpublished biomarker that predicts unfolded protein response mediated by the X-box binding protein 1 (XBP1; unpublished data). The correlations between each biomarker and the gene lists of BPA and alternatives were determined using the Running Fisher algorithm in the BaseSpace Correlation Engine as described previously (Ryan et al. 2016). The Running Fisher algorithm provides an assessment of the statistical significance of the correlation of the overlapping genes between the biomarker and each gene list. A complete description of the Running Fisher test has been published (Kupershmidt et al. 2010). The results were exported, and each p value was converted to a − log(*p* value). Negative values were used to indicate negative correlation between the biomarker and the gene list. Thresholds for significance were set at − log(*p* value) ≥ 4 for activation or ≤  − 4 for suppression based on prior studies using this threshold (Matteo et al. 2023).

### Transcriptomic point of departure analysis

BMC modeling was done as described previously (Matteo et al. 2023) in BMDExpress3. A Williams trend test (p < 0.05) and absolute fold change ≥ 1.5 was applied to filter probes. Best fit models from polynomial 2°, linear, power (power term constrained to ≥ 1), and exponential models (degrees 3 and 5) were selected for each probe based on the lowest Akaike Information Criterion. A benchmark response of 1 standard deviation was selected. Probes with the following criteria were removed: (1) having a BMC greater than the highest concentration used in the analysis after excluding cytotoxic or non-soluble concentrations; (2) mapping to more than one gene; (3) having a model fit p value < 0.1 determined by a likelihood ratio test; (4) having a BMC to BMC lower (BMCL) ratio > 20, (5) having a BMC upper (BMCU) to BMCL ratio > 40. Probes that met all the BMC filtering criteria were converted to their corresponding Entrez Identifiers. Data were further analyzed using R statistical software (version 4.3.2). Gene accumulation plots were generated by rank ordering genes fitting BMCs (lowest to highest) and plotting the BMC of each gene on the *x*-axis and rank along the *y*-axis.

Two types of tPODs were produced that we called: (1) general toxicity; and (2) ERα-specific response.

Three methods were used to derive general toxicity tPODs:

- 25th rank-ordered gene BMC: As previously described (Matteo et al. 2023), genes fitting BMCs were rank ordered from lowest to highest BMC and the BMC for the 25th gene was selected as an early transcriptomic tPOD. Confidence intervals were derived using the BMCU and BMCL as upper and lower limits, respectively.
- Lowest pathway BMC: We derived the lowest pathway tPOD by aligning genes and their associated BMCL/BMC/BMCU values to the gene sets in Reactome Pathways database (https://reactome.org/) or the Kyoto Encyclopedia of Genes and Genomes (KEGG; https://www.genome.jp/kegg/). Gene sets that contained at least three genes fitting BMCs (genes that pass all criteria in the analysis), were at least 5% populated (based on total annotated gene number), and contained at least 40 genes in the whole gene set were selected. The 5th percentile BMC of the selected gene sets were selected as tPODs.
- Lowest Consistent Response Dose (LCRD): We used an approach by Crizer and colleagues to identify the lowest concentration at which a consistent change in biologic activity begins to occur (Crizer et al. 2021).

We derived two ERα-specific tPODs by calculating (a) the median BMC and (b) the 5th percentile BMC of 50 genes that comprise a published ERα biomarker (Corton et al. 2024a, b). We first confirmed that a compound or mixture activated the ERα biomarker for at least one exposure concentration as described below (see Gene expression biomarker analysis). We further filtered by requiring a minimum of three genes with BMCs in the biomarker gene set to derive an ERα BMC. For all tPODs, confidence intervals for gene sets were estimated using a parametric bootstrap where the residuals were assumed to be normally distributed. For each gene, 100 bootstrap samples were generated followed by BMC analysis. Experiments were simulated using the bootstrapped results to obtain a BMC distribution. Experiment inclusion probabilities for each gene was estimated using the proportion of bootstrap samples that resulted in a BMC. For each simulated experiment a uniform random number between 0 and 1 was generated for each gene. If the random number was less than the gene’s inclusion probability, the gene was retained and a BMC from the list of estimated BMCs for that gene was randomly selected. The median value from each of the 10,000 simulated experiments was used to estimate the tPOD median. The 95% confidence interval was estimated using the 2.5th and 97.5th percentiles from the simulated distribution.

### Mixture modelling

Since most of the BPA alternative chemicals tested activate ERα (Matteo et al. 2023), we assumed that most would have additive effects when tested in vitro. We used the same method as previously described (Altenburger et al. 2000; Addicks et al. 2023) to predict the BMCs of mixtures of chemicals (BMC_pred_) from the BMCs of the individual components (BMC_*i*_) and their molar fractions (*p*_*i*_) in Eq. 1.

1 $${\text{BMC}}_{\text{pred}}={\left({\sum }_{i=1}^{n}\frac{{p}_{i}}{{\text{BMC}}_{i}}\right)}^{-1}$$

### Ingenuity pathway analysis

Ingenuity pathway analysis (IPA; QIAGEN, Redwood City, CA, USA) was used to identify changes to upstream regulators, canonical pathways, and disease and functions. For each chemical tested, we imported Excel files into IPA containing gene IDs (Gene Symbol and Entrez ID) for the genes that fit a BMC (i.e., passed all filtering criteria), as well as their Williams trend test *p* values from the BMC analysis, and the largest fold-changes of the gene relative to solvent controls (exported filtered data from BMDExpress3). This approach allowed us to analyze for the enrichment of genes showing robust concentration-responses to the exposures. IPA Core Analysis with a gene expression threshold of absolute fold change ≥ 1.5 and false discovery rate (FDR) adjusted *p* value ≤ 0.05 was used with the direct and indirect relationship settings based on experimental and highly predicted data. Statistical significance of the overlap (FDR-adjusted *p* value ≤ 0.05) between the data set and known targets of upstream regulators in IPA were calculated using Fisher’s exact tests. The *z* score was calculated using Fisher’s exact test based on the expected relationship for directions between upstream regulators and target genes and those observed in the data set. A *z* score of > 2 (activated) or < 2 (inhibited) was considered statistically significant. As a secondary analysis, we binned genes fitting BMCs by rank order (least to greatest BMC) set to 1–100, 101–200, and 201–300 to study specific transcriptomic changes initiated at increasing concentrations.

## Results

### Cell viability

The CellTiter-Blue Cell Viability assay was used to assess cytotoxicity after 48 h of chemical exposure. There was no evidence of cytotoxicity following exposure to any mixture or individual BPA alternative chemical (Supplementary Fig. 1).

### General transcriptomic data summary

The median read depth was approximately 2-million per sample with a median range of 89.8% mapped reads. Of 672 samples, 70 experimental samples were lost to QC, along with 28 reference RNA and lysis buffer control samples (Supplementary File 2). The total number of samples per condition is listed in Supplementary Table 1.

**Fig. 2:**
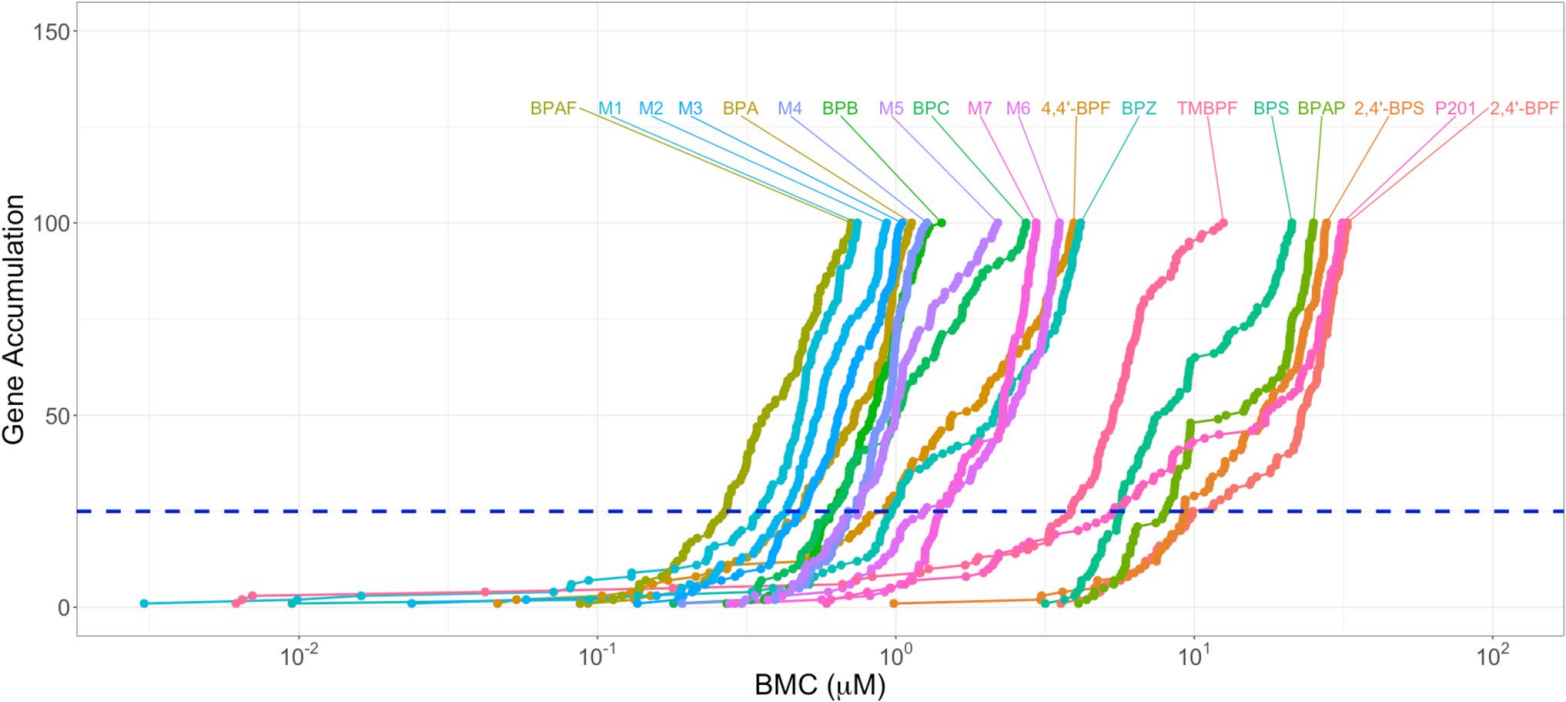
Gene accumulation plot; data were prefiltered using the Williams trend test (*p* < 0.05) and an absolute fold-change filter of ≥ 1.5, and post filtered with the following settings in BMDExpress v3: best BMD/BMDL < 20, best BMDU/BMDL < 40, and best fitPvalue >  = 0.1

The total number of genes fitting BMCs for each chemical tested is shown in Table 3. All mixtures of BPA alternatives and their individual components were transcriptionally active. Most chemicals and mixtures tested had 300–600 genes fitting BMCs. TMBPF (1191), M7 (714), and M1 (703) had the most genes fitting BMCs; P201 (205), 2,4′-BPF (200), and 2,4′-BPS (168) had the least. The gene accumulation plot shows the first 100 genes fitting BMCs for each mixture and chemical tested (Fig. 2).

**Table 3:** Comparison of transcriptomic points of departure (tPODs)

Chemical	# Genes fitting BMCs	25th Gene BMC	25th Gene BMCL/BMCU	25th Gene predicted BMC	# Genes fitting ERα biomarker	ERα 5th percentile BMC	ERα 5th percentile BMCL/BMCU	ERα 5th percentile predicted BMC
BPA	609	0.49	0.10–1.56		21	0.53	0.17–1.43	
BPAF	413	0.27	0.05–0.58		20	0.15	0.04–0.41	
BPC	562	0.61	0.19–4.54		23	0.51	0.12–1.65	
BPB	559	0.61	0.50–1.02		21	0.54	0.25–1.86	
4,4′-BPF	511	0.93	0.19–3.17		20	1.14	0.45–2.56	
BPZ	447	0.97	0.51–2.70		14	1.20	0.29–3.38	
TMBPF	1191	3.88	1.05–7.81		4			
P201	205	5.34	3.29–13.74		1			
BPS	356	5.67	3.56–13.55		12	6.96	4.13–10.0	
BPAP	265	8.03	4.99–11.6		2			
2,4′-BPS	168	9.28	5.75–9.60		0			
2,4′-BPF	200	9.89	4.57–41.21		3	36.8	22.2–106	
M1	703	0.35	0.060–1.69	0.35	18	0.08	0.013–0.36	0.23
M2	498	0.43	0.080–1.13	0.40	20	0.24	0.070–0.64	0.28
M3	527	0.48	0.10–1.02	0.47	19	0.19	0.054–0.52	0.34
M4	539	0.69	0.33–1.43	0.58	23	0.83	0.38–1.94	0.43
M5	634	0.75	0.37–4.09	0.68	24	0.79	0.39–1.32	0.51
M6	585	1.24	0.32–5.00	0.98	17	0.75	0.22–2.13	0.76
M7	714	1.40	1.17–9.27	1.40	17	1.91	1.24–2.84	1.66

tPODs were derived by prefiltering data using the Williams trend test (*p* < 0.05) and an absolute fold-change filter of ≥ 1.5 and postfiltered with the following settings in BMDExpress v3: best BMD/BMDL < 20, best BMDU/BMDL < 40, and best fitPvalue >  = 0.1; tPODs (shown in µM) representing the 25th rank ordered gene benchmark concentration (BMC) and the 5th percentile gene BMC for the estrogen receptor alpha (ERα) biomarker gene set are shown; most chemicals are shown in decreasing order of potency based on tPODs from the 25th gene BMC, followed by the mixtures

### Stress response biomarker activation

We used published transcriptomic biomarkers to explore general cellular stress responses following exposure to mixtures of BPA alternative chemicals and their individual components. The biomarkers were specific to: (a) NRF2 (Rooney et al. 2020); (b) HSF1 (Cervantes and Corton 2021); (c) MTF1 (Jackson et al. 2020); (d) AhR (Oshida et al. 2015); (e) TGx-DDI (Li et al. 2019); (f) TGx-HDACi (Cho et al. 2021); (g) NF-kB (Korunes et al. 2022); (h) HIF1 (unpublished data); (i) CP (Corton et al. 2024a, b); (j) XBP1 (unpublished data). As in our previous work (Matteo et al. 2023) we considered activation of at least two of these biomarkers within a condition as an indication of overt cellular stress (Escher et al. 2020) and thus filtered out these concentrations from subsequent BMC analyses. Supplementary File 3 provides a summary of all stress response biomarkers activated and inhibited.

Many BPA alternative chemicals and all mixtures activated the CP biomarker at the highest exposure concentrations tested: 2,4′-BPF (50 µM), 4,4′-BPF (10, 50 µM), BPA (5, 10, 50 µM), BPAF (5, 10, 50 µM), BPB (5, 10, 50 µM), BPC (5, 10, 50 µM), BPS (50 µM), BPZ (10, 50 µM), M1 (5, 10, 50 µM), M2 (5, 10, 50 µM), M3 (10, 50 µM), M4 (1, 5, 10, 50 µM), M5 (5, 10, 50 µM), M6 (10, 50 µM), M7 (10.5, 21 µM). Some chemicals inhibited the CP biomarker at exposure concentrations ≤ 1 µM: 2,4′-BPS (0.5, 1, 5 µM), BPAP (0.001, 0.5 µM), BPB (0.01, 0.1 µM), BPS (0.001, 0.01, 0.1, 0.5), BPZ (0.01, 0.1 µM), P201 (0.01, 0.1, 0.5, 1 µM). Few of the other transcriptomic biomarkers used in the study were perturbed by chemical exposure. Two chemicals inhibited the HIF1 biomarker BPB (50 µM) and BPC (10 µM), while the XBP1 biomarker was inhibited by M6 (10 µM). Based on this analysis, no treatment conditions were considered overtly cytotoxic.

### ERα biomarker activation

We used a 50 gene transcriptomic ERα biomarker (Corton et al. 2024a, b) to test the ability of mixtures of BPA alternative chemicals and their individual components to activate/inhibit ERα. Many BPA alternatives tested, including all mixtures, activated the ERα biomarker at the highest exposure concentrations tested (Supplementary Fig. 2). As well, some chemicals inhibited the ERα biomarker at exposure concentrations ≤ 1 µM (Supplementary Fig. 2). Three chemicals did not activate the ERα biomarker at any concentrations tested: 2,4′-BPS, P201, and TMBPF. See Supplementary File 3 for a detailed breakdown of ERα biomarker activity. Overall, the results show that all mixtures and most individual components activated the ERα biomarker at exposure concentrations ≥ 5 µM and some chemicals inhibited the biomarker at lower concentrations.

### Transcriptomic point of departure (tPOD) derivation

We used two approaches to generate tPODs based on either (a) general toxicological effects or (b) ERα activation (mode of action-specific).

#### Potency ranking for general toxicity

Given that transcriptomic activity other than that produced by ERα may cause adverse effects, we derived general toxicity tPODs to identify concentrations producing a ‘concerted molecular change’ (Johnson et al. 2022). The gene accumulation plot shows the rank order of the potency of the mixtures of BPA alternatives and individual chemicals based on the 25th gene BMC, from most (left on graph) to least potent (right side of graph). BPAF was the most potent chemical tested, followed by M1, and M2 (Fig. 2).

We used three approaches to identify exposure concentrations that cause a robust change in transcriptomic activity: (1) the 25th gene BMC, (2) the lowest pathway gene set 5th percentile BMC, and (3) the LCRD.

**Fig. 3:**
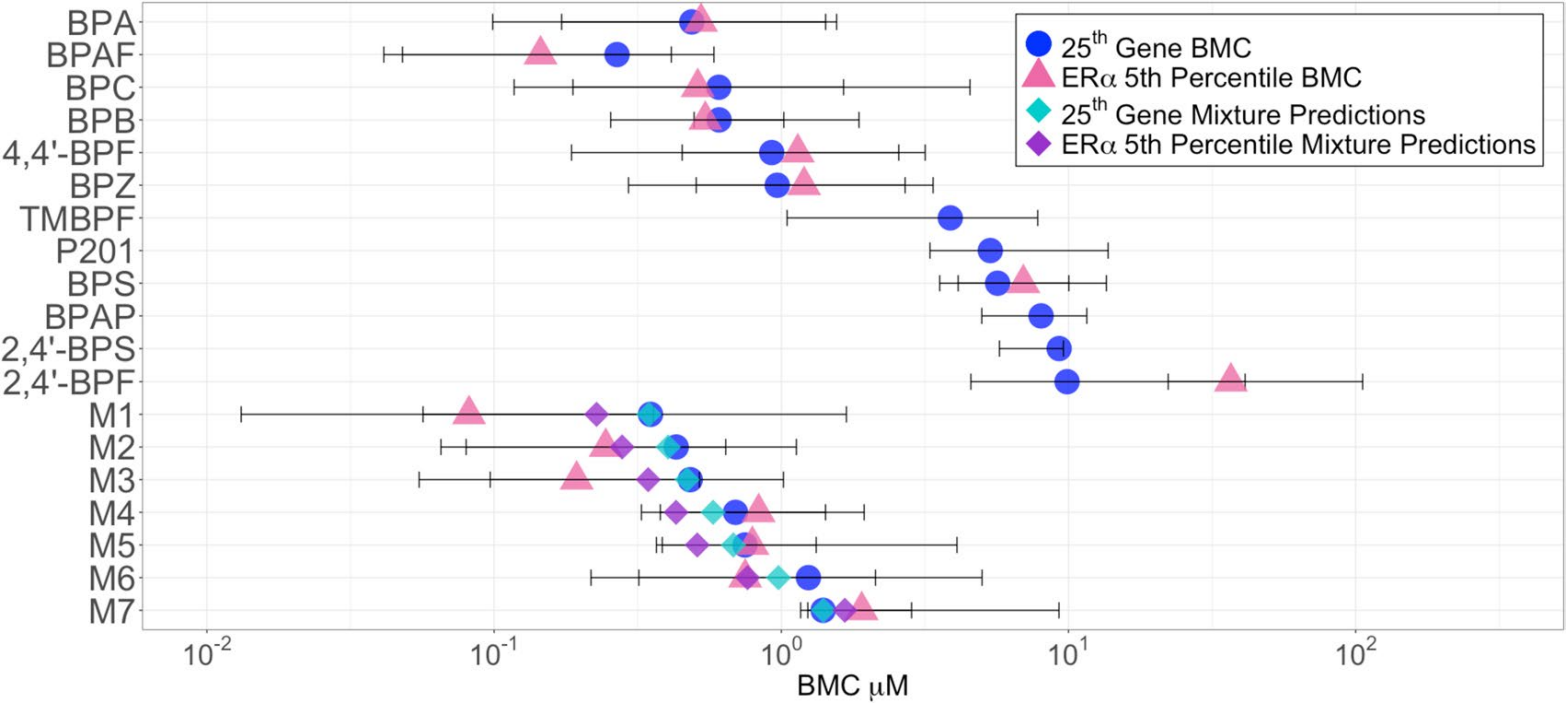
Comparison of transcriptomic points of departure (tPODs); tPODs were derived by prefiltering data using the Williams trend test (*p* < 0.05) and an absolute fold-change filter of ≥ 1.5 and postfiltered with the following settings in BMDExpress v3: best BMD/BMDL < 20, Best BMDU/BMDL < 40, and best fitPvalue >  = 0.1; tPODs (shown in µM) representing the 25th rank ordered gene benchmark concentration (BMC) and the 5th percentile gene BMC for the estrogen receptor alpha (ERα) biomarker gene set are shown; BMCL and BMCU are used for the lower and upper bounds, respectively; predictions based on additivity of individual components are shown as diamonds for the mixtures; most chemicals are shown in decreasing order of potency based on tPODs from the 25th gene BMC, followed by the mixtures

The 25th gene BMC is used to filter out genes that are within the noise range of the system and chemicals with no biologic activity, while identifying the low end of the concentration range where the transcriptome is altered (see Fig. 2). All the chemicals and mixtures tested had a 25th gene BMC, with most being between 0.1–1 µM (Table 3, Fig. 3). BPAF was the most potent chemical tested based on the 25th gene (0.27 µM), followed by M1 (0.35 µM), and M2 (0.43 µM). BPAP (5.0 µM), 2,4′-BPS (5.8 µM), and 2,4′-BPF (9.9 µM) were the least potent chemicals tested in this experiment.

The 5th percentile BMC for the lowest pathway BMC was derived because this represents an approach akin to that originally recommended by the US National Toxicology Program (National Toxicology Program 2018). The tPODs derived using this approach showed highly similar values and trends to the 25th gene BMC (Supplementary Table 2, Supplementary Fig. 4). BPAF had the lowest 5th percentile lowest pathway BMC (0.19 µM), followed by M2 (0.30 µM) and M1 (0.30 µM). BPS (6.6 µM), 2,4′-BPF (19 µM), and 2,4′-BPS (30 µM) had the highest 5th percentile lowest pathway BMCs.

The LCRD identifies an inflection point in transcriptomic activity on the accumulation curve. For most chemicals and mixtures tested, the LCRD was one order of magnitude lower than the 25th gene and the pathway BMC (Supplementary Table 2, Supplementary Fig. 4). BPAF (0.11 µM), M1 (0.17 µM), and M2 (0.18 µM) had the lowest LCRDs, while BPAP (0.96 µM), P201 (1.21 µM), and 2,4′-BPF (1.64 µM) had the highest.

##### Potency ranking based on the ERα biomarker

To explore the potencies of the BPA alternatives and mixtures in activating the ERα, we identified the genes fitting BMCs that are part of the 50 gene transcriptomic ERα biomarker. Most ERα biomarker genes were activated at higher concentrations than the 25th gene BMC (Supplementary Fig. 3). The BMCs for genes in the ERα biomarker were used to produce a 5th percentile BMC to rank ERα-specific potency of individual BPA alternatives and mixtures (Table 3, Fig. 3). Most chemicals tested had ERα 5th percentile BMCs between 0.1–1.0 µM. M1 had the lowest ERα 5th percentile BMC (0.082 µM), followed by BPAF (0.15 µM), and M3 (0.19 µM). M7 (1.9 µM), BPS (7.0 µM), and 2,4′-BPF (37 µM) had the highest ERα 5th percentile BMCs. We note that 2,4′-BPS, P201, and TMBPF did not activate the ERα biomarker and thus were not used to produce a 5th percentile ERα BMC. Two chemicals, BPAP and P201, did not meet the criteria for minimum number of genes fitting BMCs in the biomarker.

We also derived a median BMC using the 50 gene ERα biomarker (Supplementary Table 2, Supplementary Fig. 4) as a more robust central metric for potency ranking. All mixtures of BPA alternative chemicals and most of their individual components had ERα biomarker median BMCs within one order of magnitude of each other. M1 had the lowest ERα biomarker median BMC (0.50 µM), followed by BPAF (0.58 µM), and M3 (0.64 µM). BPZ (4.6 µM), BPS (22 µM), and 2,4′-BPF (37 µM) had the highest ERα biomarker median BMCs. Although the BMCs for these medians were generally higher, the potency rank order was nearly identical to the 5th percentile ERα.

### Mixture modeling

A main objective of this research was to determine if the BPA alternative chemicals interact or cause additive effects when present in mixtures. To address this, the BMCs of individual BPA alternative chemicals were used to predict their BMCs when present in mixtures using an additive model (Eq. 1). We compared the predicted mixture tPOD to the empirically derived tPOD using the 25th gene BMC, the lowest 5th percentile pathway BMC, and ERα 5th percentile BMC (Table 3, Fig. 3), as well as ERα biomarker median BMC, and the LCRD (Supplementary Table 2, Supplementary Fig. 4).

We found that most mixture BMC predictions were within the BMCL/BMCU of their experimentally derived BMCs (Table 3, Fig. 3, Supplementary Table 2, Supplementary Fig. 4). For M2–M7, the predicted tPODs were virtually identical to their empirically derived tPODs. However, there were some notable differences for M1. The ERα 5th percentile BMC was one order of magnitude lower than predicted, but still within the empirical BMCL/BMCU (Table 3, Fig. 3). Overall, these data support that mixtures of BPA alternative chemicals tested behave additively at concentrations near their BMCs.

### Ingenuity pathway analysis

To determine the primary mechanisms altered by the chemicals and mixtures, we used the genes fitting BMCs, their maximum fold changes, and their p values, in an IPA canonical pathway and upstream regulator analysis as described previously (Matteo et al. 2023). The mixtures and many of their individual chemical components perturbed similar upstream regulators and pathways, supporting common modes of action. A full list of canonical pathways and upstream regulators affected by chemical exposure is available in Supplementary File 4.

**Fig. 4:**
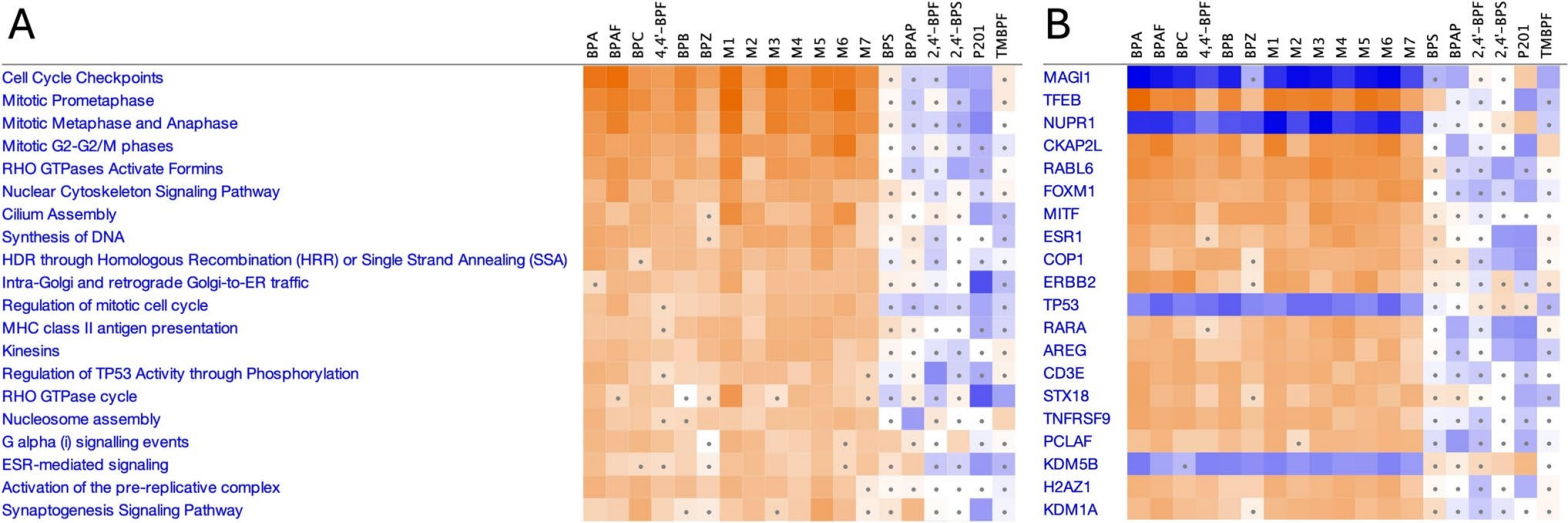
Ingenuity pathway analysis; a list of genes fitting benchmark concentration models, the Williams trend test *p* value, and the maximum fold-change were imported into IPA; filters were set to *Z* score ≥ 2.0 and p value < 0.05; orange denotes predicted activation and blue predicted inactivation (dots signify *Z* score < 2); **a** top 20 upstream regulators; **b** top 20 canonical pathways

All the mixtures of BPA alternatives tested perturbed similar upstream regulators and canonical pathways along with several of their individual components: BPA, BPAF, BPC, 4,4′-BPF, BPB, and BPZ (Fig. 4a, b). Most of the pathways predicted to be activated were associated with increased cellular proliferation and estrogen-related signaling (Fig. 4a). Importantly, many of these same chemicals activated the CP biomarker (Supplementary File 3). All mixtures and most individual chemicals were predicted to inhibit MAGI1, NUPR1, and TP53 (Fig. 4b). Further, these same chemicals were predicted to activate TFEB, CKAP2L, RABL6, FOXM1, MITF, COP1, and ERBB2. In contrast, BPS, BPAP, 2,4′-BPF, 2,4′-BPS, P201, and TMBPF mostly caused opposite effects relative to the other chemicals tested, but these typically did not reach statistical significance (Fig. 4b). All mixtures and most individual BPA alternative chemicals that were predicted to activate ESR1 were identified as ERα activators using the transcriptomic biomarker (Supplementary File 3). Some chemicals that were identified as inhibitors of the ERα biomarker, such as 2,4′-BPS and P201, were also predicted to inhibit ESR1 using this IPA-based approach.

We rank ordered and binned the genes fitting BMCs into three bins to explore the mechanisms associated with transcriptional changes occurring in different BMC ranges:

- Bin 1: genes BMCs ranked 1–100 (lowest BMCs, Supplementary Fig. 5)
- Bin 2: genes BMCs ranked 101–200 (medium BMCs, Supplementary Fig. 6)
- Bin 3: genes BMCs ranked 201–300 (high BMCs, Supplementary Fig. 7)

For Bin 1, most of the mixtures, along with BPA, BPAF, and BPC, were enriched for pathways and upstream regulators associated with cellular proliferation and the estrogen receptor (Supplementary Fig. 5). Bin 2 gene sets were over-represented by the mixtures, with fewer genes fitting BMCs leading to enriched pathways for BPA, BPAF, and BPC (Supplementary Fig. 6). Interestingly, P201 inversely modulated the genes enriched in this bin relative to most of the other chemicals in Bin 2. In Bin 3, most mixtures, as well as 4,4′-BPF, BPB, and BPZ were enriched in similar pathways and upstream regulators associated with cellular proliferation and the estrogen receptor (Supplementary Fig. 7). Finally, the genes with BMCs for the chemicals 2,4′-BPS (168 genes fitting BMCs; Bins 1 and 2 only), 2,4′-BPF (200 genes fitting BMCs; Bins 1 and 2 only), and TMBPF (1191 genes fitting BMCs) were not enriched in the same pathways and upstream regulators as the other chemicals and mixtures.

## Discussion

Given the growing number of BPA replacements and human exposure to these compounds in mixtures, we tested whether mixtures of BPA alternatives have additive effects in MCF-7 cells using HTTr. We previously determined the potency of BPA and 15 alternative chemicals in MCF-7 cells (Matteo et al. 2023) and used the most potent ERα active chemicals from that study to design six equimolar mixtures. We produced a seventh complex mixture using the relative proportions of BPA alternatives detected in human urine samples (Gys et al. 2020). We did BMC modeling on gene expression data to derive ERα-specific and general toxicity tPODs. We applied a predictive model previously used by our lab to test for additive effects in vitro (Addicks et al. 2023). All the mixtures in our study activated ERα based on a transcriptomic biomarker analysis. In addition, using tPODs derived from the ERα biomarker and general toxicity methods it was observed that all predicted mixture tPODs were nearly identical to their experimentally derived tPODs. Genes fitting BMCs analyzed with IPA revealed that mixtures perturbed similar upstream regulators and canonical pathways as their individual components. Collectively, our data support additive effects of BPA alternative chemicals in vitro through both their ERα activities and alternative mechanisms of toxicity that they perturb.

All mixtures and most of the BPA alternatives tested were identified as ERα active. Importantly, equimolar mixtures, as well as the complex mixture tested, activated ERα at similar concentrations as BPA. This aligns with previous reports of mixtures of BPA alternatives (that include BPA) activating ERα in vitro (Bermudez et al. 2010; Park et al. 2020; Lee et al. 2023). Interestingly, three chemicals that we previously determined to be ERα agonists (2,4′-BPS, BPAP, and P201) (Matteo et al. 2023), inhibited the 50 gene ERα biomarker in this study. Further, several BPA alternatives tested inhibited the 50 gene ERα biomarker at concentrations below 1 µM despite being ERα active above this concentration. Together, these data suggest that BPA alternatives may have nonmonotonic effects, wherein low exposure concentrations (< 1 µM) inhibit ERα, which aligns with the low dose effects of BPA and other nuclear hormone endocrine disruptors (Vandenberg et al. 2012; Shioda et al. 2013). We also used several transcriptomic biomarkers to detect concentrations at which cells would be overtly stressed by chemical exposure, alongside an established benchtop assay for cell viability. No conditions were cytotoxic in this study, in line with previous data from our lab (Matteo et al. 2023). Our data demonstrate that mixtures of BPA alternatives activate ERα at similar concentrations as BPA and that some alternatives may inhibit ERα at low exposure concentrations.

Equimolar and human relevant mixtures of BPA alternative chemicals tested had additive effects in MCF-7 breast cancer cells. Across all tPODs derived in this study, the predicted and empirical BMCs were within an order of magnitude, supporting the use of concentration addition models for assessing mixtures of BPA alternatives in vitro. Others have previously used transcription-based assays to predict additive ERα transactivation by mixtures of BPA alternatives (Lee et al. 2023; Bermudez et al. 2010). Moreover, another group exposed MCF-7 cells to an equimolar mixture of BPA alternatives (0.35 µM) and detected almost no changes in transcriptomic activity using HTTr after 24 h exposure, despite causing significant biologic activity in MCF-10 breast epithelial cells that lack ERα expression (Mesnage et al. 2022). We also observed very few differentially expressed genes in MCF-7 cells using a mixture of similar composition (Mix 7) at concentrations below 1 µM (data not shown). In both studies, the mixtures were mostly composed of ERα active BPA alternatives, which is the primary mode of action of BPA and many alternative chemicals in MCF-7 cells. It is possible that exposure to mixtures of BPA alternatives at concentrations below 1 µM may not be sufficient to cause robust transcriptional activity in MCF-7 cells. These data support the use of HTTr data to test for chemical additivity in vitro and that mixtures of BPA alternatives have additive effects in ERα positive breast cancer cells. Further research of the effects of exposure to BPA alternative chemicals in physiologically relevant systems like normal breast epithelial cells is warranted.

We used several tPODs to rank BPA alternative chemicals based on toxicological potency in vitro. The 25th gene BMC tPOD was used to highlight early changes to the transcriptome caused by chemical exposure and rank chemicals for overall toxicological potency. We also employed a distribution-based approach to derive a conservative gene set tPOD to capture relevant biologic activity as previously described (Reardon et al. 2023). Further, an LCRD was used to determine the lowest concentration associated with significant transcription. All tPODs produced similar potency rankings and most were within one order of magnitude of each other. BPAF, a fluorinated alternative, was the most potent chemical tested overall, followed by M1, and M2. In line with additive effects, mixtures containing the most potent individual chemicals had the lowest tPODs. BPA was the second most potent individual chemical tested, followed by BPC, similar to our previous HTTr potency ranking exercise (Matteo et al. 2023). Interestingly, TMBPF, a non-estrogenic alternative to BPA (Soto et al. 2017), caused the most transcriptional activity of all chemicals tested, despite being less potent than many chemicals tested. Overall, both general and ERα tPODs aligned to produce similar potency rankings, with mixtures of BPA alternatives among the most potent chemicals tested. These data support the use tPODs to determine the potency of chemical mixtures in vitro.

All genes fitting BMCs were analyzed using IPA to identify potential modes of action of mixtures of BPA alternatives in MCF-7 cells. In line with our previous study (Matteo et al. 2023), many individual BPA alternatives perturbed similar upstream regulators including ESR1, RARA, and COP1. Moreover, mixtures and most of their individual components affected similar upstream regulators and pathways associated with loss of cell cycle control, supporting additive effects in breast cancer cells. Specifically, all mixtures tested were predicted to inhibit tumor suppressor genes like P53 (Lim et al. 2009) and MAGI1 (Wörthmüller et al. 2023). Interestingly, NUPR1, a gene associated with tamoxifen resistance in breast cancer cells (Wang et al. 2021), was predicted to be inhibited in IPA. Since NUPR1 interacts with ESR1 (Wang et al. 2021), it is possible that exposure to mixtures of BPA alternatives inhibited this pathway. Further, all mixtures were predicted to activate genes associated with breast cancer tumor progression like TFEB (Wang et al. 2023), RABL6 (Li et al. 2013), FOXM1 (Ziegler et al. 2019), MITF (Zhang et al. 2024), and CKAP2L (dos Santos et al. 2022). When we binned genes according to their BMC rank order, we found that most of the affected upstream regulators and pathways were within the first 200 genes fitting BMCs. This suggests that for most chemicals tested, exposure to higher concentrations of mixtures of BPA alternatives caused non-specific transcriptomic activity. Finally, despite having the most genes fitting BMCs, TMBPF perturbed the fewest upstream regulators and pathways, suggesting that it caused non-specific biologic activity in MCF-7 cells at all concentrations tested. Collectively, these data support that mixtures of BPA alternatives have additive effects and dysregulate cellular proliferation in vitro.

In summary, our HTTr data add to the weight of evidence that mixtures of BPA alternative chemicals have additive effects in vitro. Transcriptomic biomarker analysis revealed that all mixtures and most BPA alternatives tested activated ERα at similar concentrations. General toxicity and ERα-related tPODs produced comparable potency rankings, with BPAF as the most potent chemical overall and ERα activator tested, in line with previous data from our lab and others. All mixtures and most individual chemicals had common modes of action via dysfunctional cellular proliferation and ERα-related pathways. Further research on the effects of individual BPA alternatives and mixtures thereof in physiologically relevant systems, such as normal breast epithelial cells, is warranted. Overall, these data support the use of HTTr to test mixtures as part of hazard characterization for human health risk assessment.

## Supplementary Information

Below is the link to the electronic supplementary material.

Supplementary file1 (DOCX 11780 KB)

Supplementary file2 (XLSX 30 KB)

Supplementary file3 (XLSX 161 KB)

Supplementary file4 (XLSX 22 KB)

Supplementary file5 (XLSX 126 KB)

## Data Availability

Data are available through the NCBI Gene Expression Omnibus with GEO accession GSE276869.
