# Supplementary material for: High-throughput transcriptomics analysis of equipotent and human relevant mixtures of BPA alternatives reveal additive effects in vitro
Source: Arch Toxicol. 2025 Jun 18;99(9):3707–19. doi: 10.1007/s00204-025-04110-3 (PMC12408750; doi:10.1007/s00204-025-04110-3)
Supplement: Supplementary file 1 — Supplementary file1 (DOCX 11780 KB) [file 204_2025_4110_MOESM1_ESM.docx]

High-throughput transcriptomics analysis of equipotent and human relevant mixtures of BPA alternatives reveal additive effects *in vitro*

**Authors**: Geronimo Matteo^1,2^, Eunnara Cho^1^, Marc Rigden^1^, David C. Eickmeyer^2^, Lauren M. Bradford^1^, Matthew J. Meier^1^, Andrew Williams^1^, J. Christopher Corton^3^, Carole L Yauk^2^*, Ella Atlas^1,4^* (Corresponding Authors)

^1^Environmental Health Science and Research Bureau, Health Canada

^2^Dept. of Biology, University of Ottawa

^3^Center for Computational Toxicology and Exposure, US Environmental Protection Agency

^4^Dept. of Biochemistry, University of Ottawa.

* To whom correspondence should be addressed. E-mail: ella.atlas@hc-sc.gc.ca and carole.yauk@uottawa.ca

**Supplementary Tables**

**Supplementary Table 1** Total number of replicates per condition included in the analysis

| Chemical | Concentration (µM) | | | | | | | | | | | | | |
| --- | --- | --- | --- | --- | --- | --- | --- | --- | --- | --- | --- | --- | --- | --- |
|  | 0.001 | 0.01 | 0.1 | 0.5 | 0.66 | 1 | 1.31 | 2.62 | 5 | 5.25 | 10 | 10.5 | 21 | 50 |
| 2,4'-BPF | 3 | 3 | 4 | 4 |  | 4 |  |  | 3 |  | 4 |  |  | 4 |
| 2,4'-BPS | 3 | 2 | 3 | 4 |  | 4 |  |  | 2 |  | 3 |  |  | 2 |
| 4,4'-BPF | 3 | 3 | 3 | 3 |  | 3 |  |  | 3 |  | 2 |  |  | 2 |
| BPA | 4 | 4 | 4 | 4 |  | 3 |  |  | 3 |  | 4 |  |  | 3 |
| BPAF | 4 | 3 | 4 | 4 |  | 3 |  |  | 2 |  | 4 |  |  | 3 |
| BPAP | 4 | 4 | 4 | 4 |  | 4 |  |  | 4 |  | 4 |  |  | 4 |
| BPB | 3 | 4 | 3 | 4 |  | 3 |  |  | 4 |  | 3 |  |  | 4 |
| BPC | 4 | 4 | 3 | 4 |  | 4 |  |  | 4 |  | 4 |  |  | 3 |
| BPS | 4 | 4 | 4 | 4 |  | 4 |  |  | 4 |  | 3 |  |  | 4 |
| BPZ | 4 | 3 | 3 | 3 |  | 4 |  |  | 4 |  | 4 |  |  | 4 |
| M1 | 3 | 4 | 4 | 4 |  | 3 |  |  | 4 |  | 4 |  |  | 4 |
| M2 | 4 | 4 | 4 | 4 |  | 4 |  |  | 4 |  | 4 |  |  | 4 |
| M3 | 4 | 4 | 4 | 4 |  | 4 |  |  | 4 |  | 4 |  |  | 3 |
| M4 | 4 | 3 | 4 | 4 |  | 4 |  |  | 4 |  | 4 |  |  | 2 |
| M5 | 4 | 4 | 4 | 4 |  | 4 |  |  | 3 |  | 3 |  |  | 3 |
| M6 | 4 | 4 | 4 | 4 |  | 4 |  |  | 4 |  | 3 |  |  | 4 |
| M7 |  |  |  |  | 4 |  | 4 | 3 |  | 3 |  | 4 | 4 |  |
| P201 | 3 | 3 | 3 | 4 |  | 3 |  |  | 3 |  | 3 |  |  | 3 |
| TMBPF |  | 3 | 2 |  |  | 3 |  |  | 4 |  | 3 |  |  | 3 |

**Supplementary Table 2** Comparison of transcriptomic points of departure (tPODs) derived from whole transcriptome analysis of MCF-7 cells (n = 2 – 4 per concentration) exposed to seven mixtures of bisphenol A (BPA) alternatives and 12 individual chemicals at a range of concentrations (0.001 – 50 µM) for 48 h

| Chemical | Lowest Pathway 5th Percentile BMC | Lowest Pathway 5th Percentile BMCL - BMCU | Predicted Lowest Pathway 5th Percentile BMC | # Genes fitting ERα biomarker | ERα Median BMC | ERα Median BMCU - BMCL | Predicted ERα Median BMC | LCRD | LCRD BMCL - BMCU | Predicted LCRD |  |
| --- | --- | --- | --- | --- | --- | --- | --- | --- | --- | --- | --- |
|  |  |  |  |  |  |  |  |  |  |  |  |
| BPA | 0.23 | 0.0052 - 0.49 |  | 21 | 2.73 | 2.03 - 4.15 |  | 0.18 | 0.091 - 0.32 |  |  |
| BPAF | 0.12 | 0.014 - 0.23 |  | 20 | 0.58 | 0.19 - 1.29 |  | 0.11 | 0.055 - 0.17 |  |  |
| BPC | 0.21 | 0.0043 - 0.48 |  | 23 | 2.90 | 1.55 - 5.39 |  | 0.21 | 0.11 - 0.34 |  |  |
| BPB | 0.32 | 0.015 - 0.54 |  | 21 | 3.67 | 2.61 - 6.38 |  | 0.24 | 0.14 - 0.37 |  |  |
| 4,4'-BPF | 0.18 | 0.00089 - 0.91 |  | 20 | 3.48 | 2.40 - 6.16 |  | 0.41 | 0.12 - 0.77 |  |  |
| BPZ | 0.51 | 0.0085 - 0.91 |  | 14 | 4.63 | 3.11 - 9.07 |  | 0.38 | 0.17 - 0.54 |  |  |
| TMBPF | 0.07 | 0.00062 - 1.81 |  | 4 |  |  |  | 0.45 | 0.096 - 1.56 |  |  |
| P201 | 2.48 | 0.021 - 5.47 |  | 1 |  |  |  | 1.21 | 0.51 - 2.77 |  |  |
| BPS | 0.98 | 0.040 - 3.48 |  | 12 | 22.27 | 15.7 - 38.0 |  | 0.93 | 0.49 - 2.90 |  |  |
| BPAP | 0.99 | 0.024 - 2.59 |  | 2 |  |  |  | 0.96 | 0.50 - 2.69 |  |  |
| 2,4'-BPS | 1.02 | 0.014 - 3.18 |  | 0 |  |  |  | 0.93 | 0.49 - 2.23 |  |  |
| 2,4'-BPF | 1.31 | 0.020 - 3.28 |  | 3 | 36.8 | 22.2 - 106 |  | 1.64 | 0.53 - 2.76 |  |  |
| M1 | 0.18 | 0.0039 - 0.42 | 0.16 | 18 | 0.50 | 0.22 - 1.11 | 0.96 | 0.14 | 0.073 - 0.24 | 0.14 |  |
| M2 | 0.20 | 0.017 - 0.48 | 0.17 | 20 | 0.81 | 0.24 - 2.24 | 1.24 | 0.17 | 0.092 - 0.28 | 0.15 |  |
| M3 | 0.20 | 0.014 - 0.40 | 0.17 | 19 | 0.64 | 0.22 - 1.63 | 1.48 | 0.19 | 0.10 - 0.29 | 0.18 |  |
| M4 | 0.28 | 0.011 - 0.56 | 0.21 | 23 | 1.94 | 1.16 - 4.69 | 1.84 | 0.22 | 0.12 - 0.36 | 0.22 |  |
| M5 | 0.24 | 0.0065 - 0.52 | 0.24 | 24 | 3.50 | 2.43 - 6.01 | 2.18 | 0.23 | 0.12 - 0.38 | 0.25 |  |
| M6 | 0.28 | 0.0021 - 0.66 | 0.33 | 17 | 4.09 | 2.82 - 7.32 | 3.23 | 0.33 | 0.16 - 0.51 | 0.34 |  |
| M7 | 0.32 | 0.00029 - 1.05 | 0.32 | 17 | 3.95 | 2.56 - 5.59 | 5.73 | 0.37 | 0.15 - 0.69 | 0.58 |  |

tPODs were derived by prefiltering data using the Williams trend test (p < 0.05) and an absolute fold-change filter of ≥ 1.5; data were also postfiltered with the following settings in BMDExpress v3: Best BMD/BMDL < 20, Best BMDU/BMDL < 40, and Best fitPvalue >= 0.1; tPODs (shown in µM) representing the 5^th^ percentile gene BMC for the lowest pathway (at least 3 genes and 5% of pathway), the median gene benchmark concentration (BMC) for the estrogen receptor alpha (ERα) biomarker gene set, and the lowest consistent response dose are shown; the ERα tPOD is based on at least one concentration yielding an agonist or antagonist call and at least 3 genes producing BMCs; BPA is shown at the top and then most chemicals are shown in decreasing order of potency based on tPODs from the 25th gene BMC, followed by the mixtures

**Supplementary Figures**


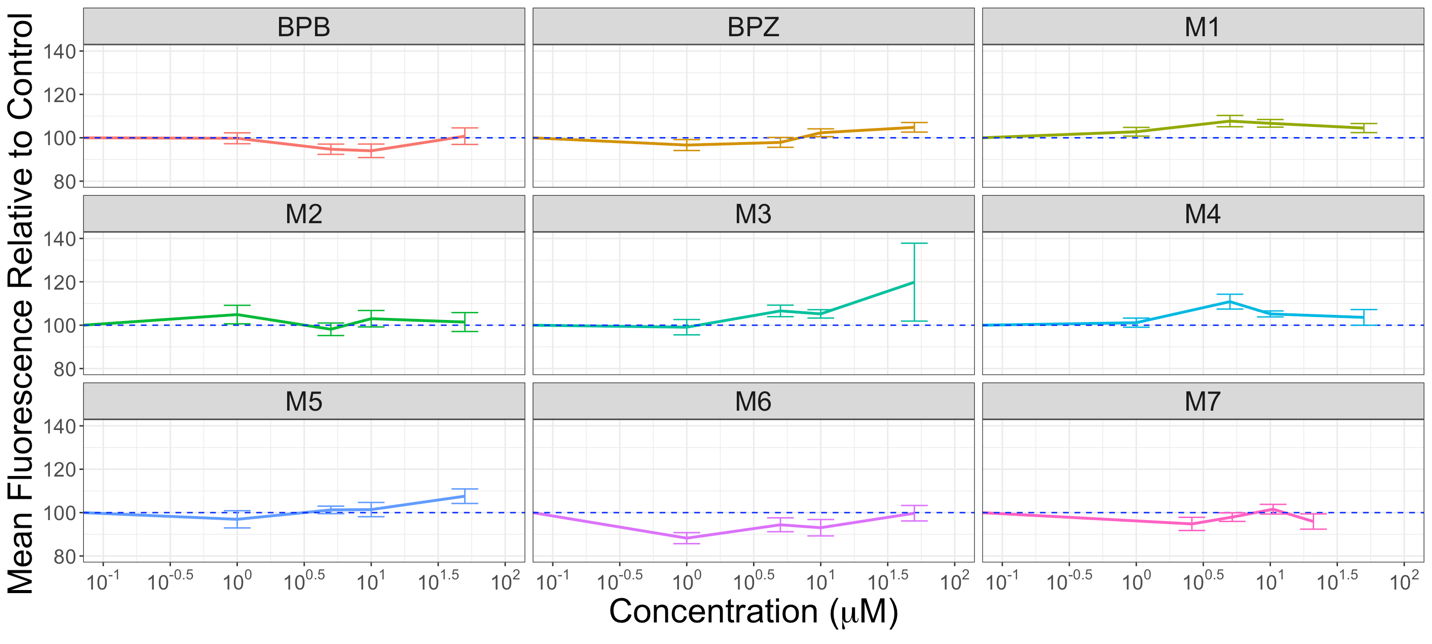
**Supplementary** **Fig. 1** Cell viability using a CellTiter-Blue Cell Viability Assay; MCF-7 cells (n = 2 – 4) were exposed to seven mixtures of bisphenol A (BPA) alternative chemicals and individual chemicals at a range of concentrations (0.001 – 50 µM) for 48 hours and are compared to their respective control (dimethyl sulfoxide; 0.1%) samples; first row of chemicals from left to right: BPB, BPZ, M1; second row: M2, M3, M4; third row: M5, M6, M7; blue horizontal dotted line denotes 100% cell viability relative to controls; there were no significant declines in viability relative to controls


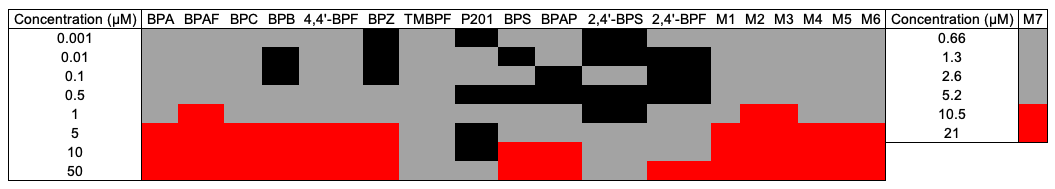


**Supplementary** **Fig. 2** Summary of estrogen receptor alpha transcriptomic biomarker activity of MCF-7 cells exposed to seven mixtures of bisphenol A (BPA) alternative chemicals and 12 individual chemicals at a range of concentrations (0.001 – 50 µM) for 48 hours; the biomarker was activated (red) or inhibited (black) based on thresholds for significance −log(p-value) ≥4 or ≤−4


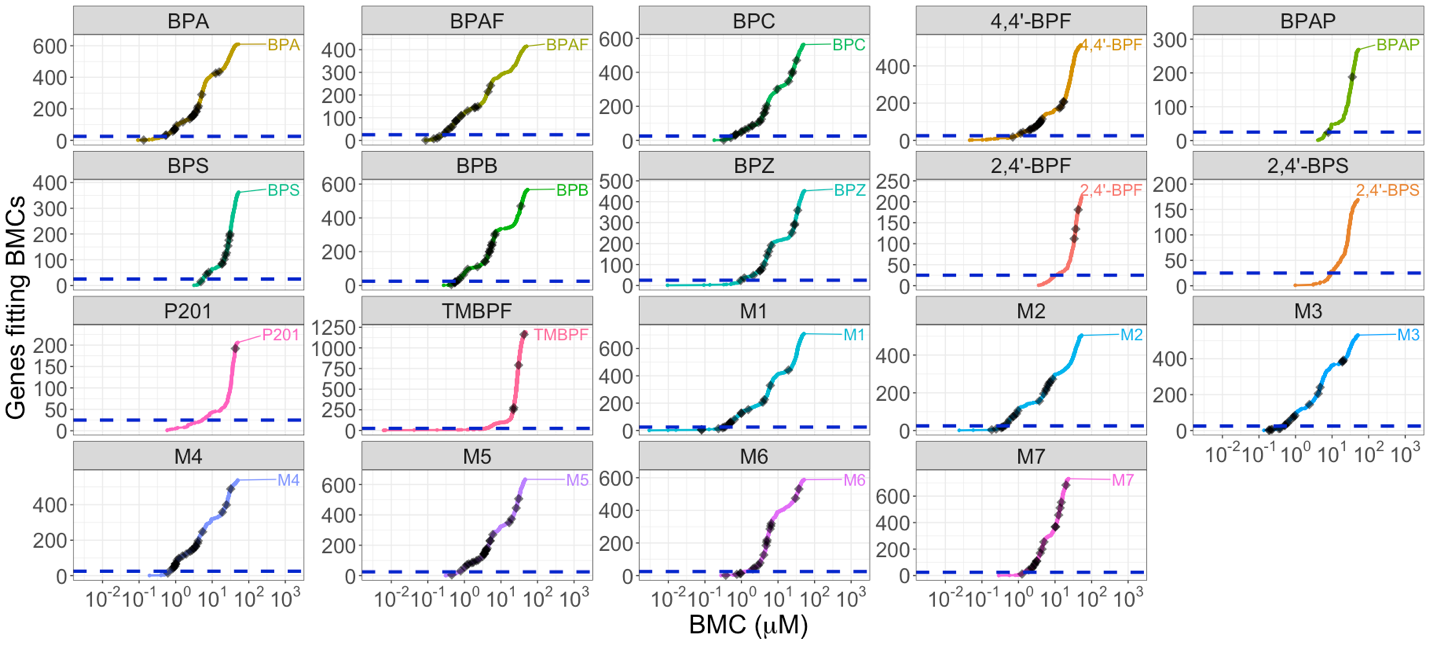


**Supplementary** **Fig. 3** Gene accumulation plot of MCF-7 cells (n = 2 – 4 per concentration) exposed to seven mixtures of bisphenol A (BPA) alternatives and 12 individual chemicals at a range of concentrations (0.001 – 50 µM) for 48 h; genes fitting the 50 gene estrogen receptor alpha biomarker are overlaid (black diamonds); the blue dashed line indicates the 25^th^ ranked gene; data were prefiltered using the Williams trend test (p < 0.05) and an absolute fold-change filter of ≥ 1.5, and post filtered with the following settings in BMDExpress v3: Best BMD/BMDL < 20, Best BMDU/BMDL < 40, and Best fitPvalue >= 0.1; first row of chemicals from left to right: BPA, BPAF, BPC, 4,4’-BPF, BPAP; second row: BPS, BPB, BPZ, 2,4’-BPF, 2,4’-BPS; third row: P201, TMBPF, M1, M2, M3; fourth row: M4, M5, M6, M7


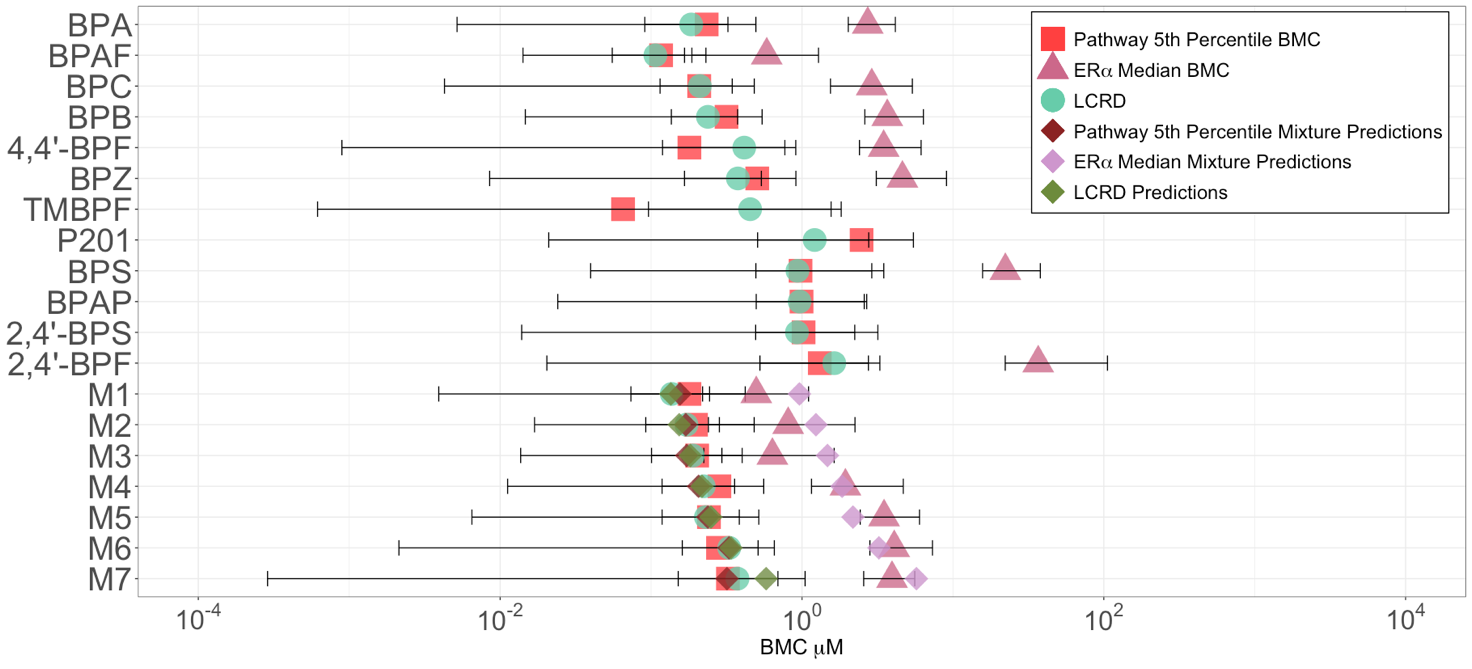


**Supplementary** **Fig. 4** Comparison of transcriptomic points of departure (tPODs) derived from whole transcriptome analysis of MCF-7 cells (n = 2 – 4 per concentration) that were exposed to seven mixtures of bisphenol A (BPA) alternatives and 12 individual chemicals at a range of concentrations (0.001 – 50 µM) for 48 h; tPODs were derived by prefiltering data using the Williams trend test (p < 0.05) and an absolute fold-change filter of ≥ 1.5; data were also postfiltered with the following settings in BMDExpress v3: Best BMD/BMDL < 20, Best BMDU/BMDL < 40, and Best fitPvalue >= 0.1; tPODs (shown in µM) representing the lowest pathway 5^th^ percentile benchmark concentration (BMC), median gene BMC for the estrogen receptor alpha biomarker gene set, and the lowest consistent response dose are shown; the ERα tPOD is based on at least one concentration yielding an agonist or antagonist call and at least 3 genes; BMCL and BMCU are used for the lower and upper bounds, respectively; the reference chemical BPA is shown at the top; most chemicals are shown in decreasing order of potency based on tPODs from the 25th gene BMC, followed by the mixtures; predictions based on additivity of individual components are shown as diamonds for the mixtures


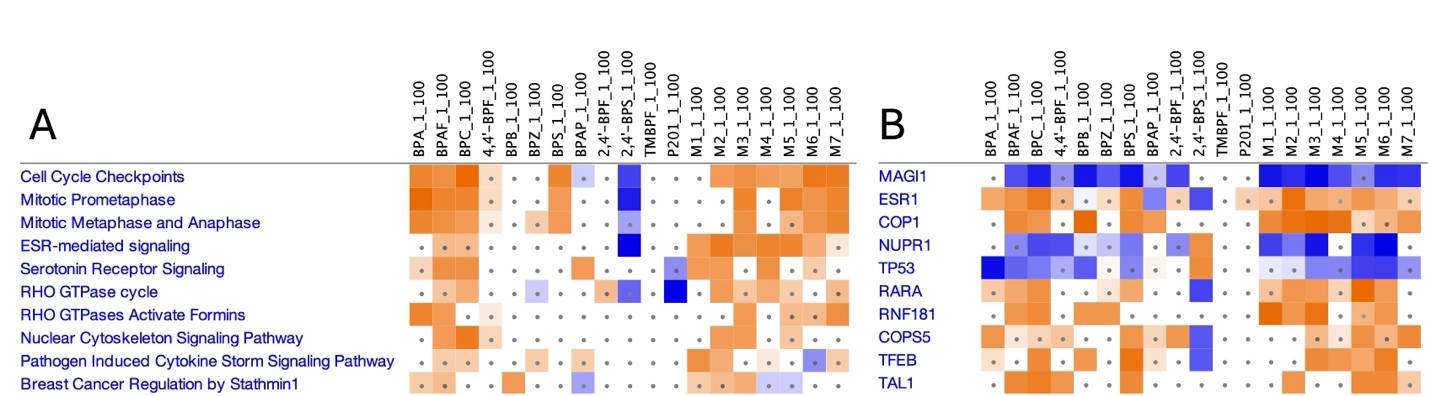


**Supplementary** **Fig. 5** Ingenuity Pathway Analysis of genes fitting models rank order 1 – 100 (Bin 1) in MCF-7 cells (n = 2 – 4 per concentration); cells were exposed to seven mixtures of bisphenol A (BPA) alternatives and 12 individual chemicals at a range of concentrations (0.001 – 50 µM) for 48 h; genes fitting models were rank ordered from least to greatest based on benchmark concentration; a list of genes fitting models with rank order 1 – 100 (inclusive), the Williams trend test p value, and the maximum fold-change were imported into IPA for this comparison; orange denotes predicted activation and blue predicted inactivation (dots signify Z-score < 2); filters were set to Z-score ≥ 2.0 and p-value < 0.05; (a) top 10 upstream regulators (b) top 10 canonical pathways


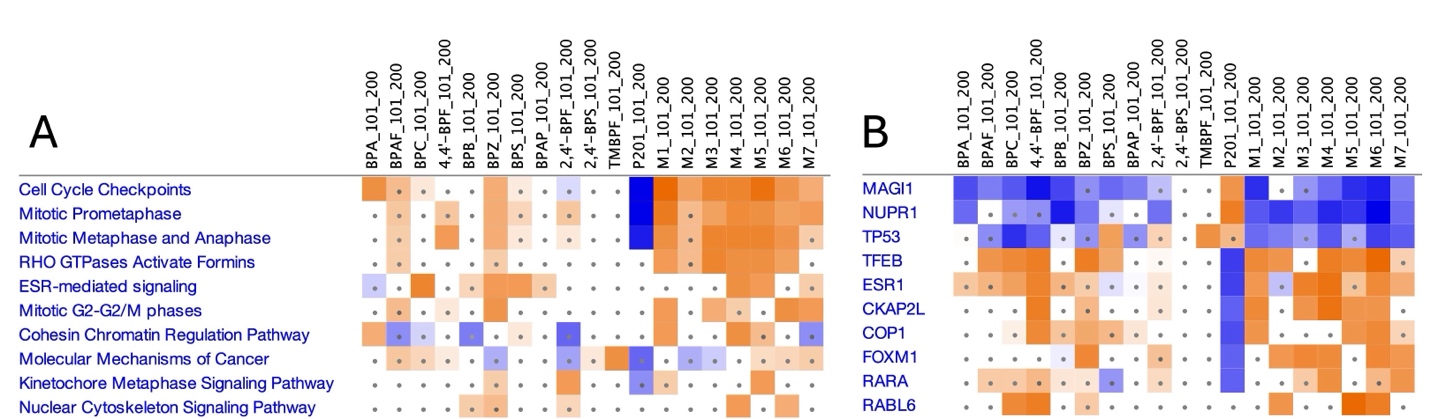


**Supplementary** **Fig. 6** Ingenuity Pathway Analysis of genes fitting models rank order 101 – 200 (Bin 2) in MCF-7 cells (n = 2 – 4 per concentration); cells were exposed to seven mixtures of bisphenol A (BPA) alternatives and 12 individual chemicals at a range of concentrations (0.001 – 50 µM) for 48 h; genes fitting models were rank ordered from least to greatest based on benchmark concentration; a list of genes fitting models with rank order 1 – 100 (inclusive), the Williams trend test p value, and the maximum fold-change were imported into IPA for this comparison; orange denotes predicted activation and blue predicted inactivation (dots signify Z-score < 2); filters were set to Z-score ≥ 2.0 and p-value < 0.05; (a) top 10 upstream regulators (b) top 10 canonical pathways


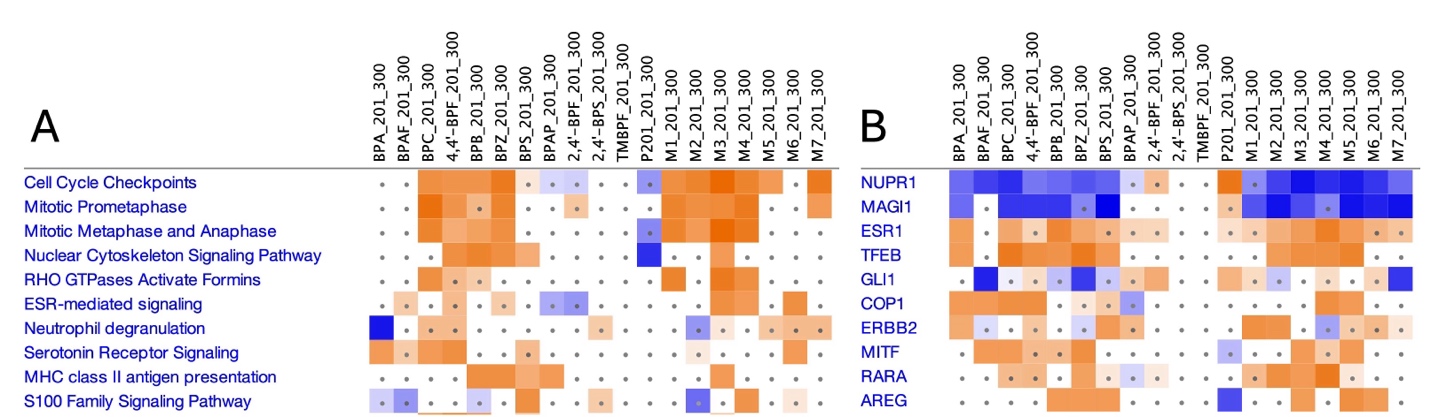


**Supplementary** **Fig. 7** Ingenuity Pathway Analysis of genes fitting models rank order 201 – 300 (Bin 3) in MCF-7 cells (n = 2 – 4 per concentration); cells were exposed to seven mixtures of bisphenol A (BPA) alternatives and 12 individual chemicals at a range of concentrations (0.001 – 50 µM) for 48 h; genes fitting models were rank ordered from least to greatest based on benchmark concentration; a list of genes fitting models with rank order 1 – 100 (inclusive), the Williams trend test p value, and the maximum fold-change were imported into IPA for this comparison; orange denotes predicted activation and blue predicted inactivation (dots signify Z-score < 2); filters were set to Z-score ≥ 2.0 and p-value < 0.05; (a) top 10 upstream regulators (b) top 10 canonical pathways

**Supplementary Files**

Supplementary File 1 List of TempO-Seq attenuators used

Supplementary File 2 List of samples removed due to QA/QC

Supplementary File 3 Summary of stress response and ERα biomarker activity

Supplementary File 4 IPA canonical pathways, upstream regulators for all genes fitting models
